# Incorporation of GM-CSF or CD40L Enhances the Immunogenicity of Hantaan Virus-Like Particles

**DOI:** 10.3389/fcimb.2016.00185

**Published:** 2016-12-20

**Authors:** Lin-Feng Cheng, Fang Wang, Liang Zhang, Lan Yu, Wei Ye, Zi-Yu Liu, Qi-Kang Ying, Xing-An Wu, Zhi-Kai Xu, Fang-Lin Zhang

**Affiliations:** Department of Microbiology, Fourth Military Medical UniversityXi'an, China

**Keywords:** Hantaan virus, virus-like particles, baculovirus expression system, GM-CSF, CD40L

## Abstract

A safe and effective Hantaan virus (HTNV) vaccine is highly desirable because HTNV causes an acute and often fatal disease (hemorrhagic fever with renal syndrome, HFRS). Since the immunity of the inactivated vaccine is weak and the safety is poor, HTNV virus-like particles (VLPs) offer an attractive and safe alternative. These particles lack the viral genome but are perceived by the immune system as virus particles. We hypothesized that adding immunostimulatory signals to VLPs would enhance their efficacy. To accomplish this enhancement, we generated chimeric HTNV VLPs containing glycosylphosphatidylinositol (GPI)-anchored granulocyte macrophage colony-stimulating factor (GM-CSF) or CD40 ligand (CD40L) and investigated their biological activity *in vitro*. The immunization of mice with chimeric HTNV VLPs containing GM-CSF or CD40L induced stronger humoral immune responses and cellular immune responses compared to the HTNV VLPs and Chinese commercial inactivated hantavirus vaccine. Chimeric HTNV VLPs containing GM-CSF or CD40L also protected mice from an HTNV challenge. Altogether, our results suggest that anchoring immunostimulatory molecules into HTNV VLPs can be a potential approach for the control and prevention of HFRS.

## Introduction

Hantaan virus (HTNV) belongs to the *Hantavirus* genus of the *Bunyaviridae* family and is a rodent-borne, enveloped RNA virus that is composed of three single-stranded RNA segments, L (large), M (medium), and S (small), that encode an RNA polymerase, the glycoproteins (GPs) Gn and Gc, and a nucleocapsid protein (NP), respectively, (Schmaljohn and Hjelle, 1997; Khaiboullina et al., 2005). HTNV causes a febrile illness in humans, namely, hemorrhagic fever with renal syndrome (HFRS). There are more than 100,000 cases per year, primarily in Asia, with a case-fatality rate of 10–15% (Zeier et al., 2005; Hooper et al., 2006).

Given the severe clinical complications and widespread geographical distribution of the HTNV infection, the prevention of this infection has been one of the major concerns in the public health field. Because there are no drugs against the HTNV infection, vaccination remains the most desirable option for disease prevention. Inactivated vaccines have contributed to a steady drop in hospital admissions for HFRS (Schmaljohn, 2009). Nevertheless, inactivated vaccines seldom elicit protective cellular responses despite its neutralizing activity, and there are no studies reporting that it could establish long-term memory immunity (Zhang et al., 2007; Song et al., 2016). Safety is another major obstacle of inactivated vaccines because it may contain some infectious particles. Therefore, approaches to HTNV vaccine development that are based on recombinant vectors, recombinant proteins, or multiprotein assemblies, such as virus-like particles (VLPs), have been proposed (Kamrud et al., 1999; Li et al., 2007, 2010, 2012, 2013).

Many viral structural proteins, including HTNV, have the intrinsic ability to assemble into VLPs that are similar in size to viruses but lack the viral genetic materials. Some VLP-based vaccines have already been licensed and commercialized. The prophylactic human vaccines against hepatitis B virus (HBV) and human papilloma virus (HPV), both based on VLPs derived from these viruses, have been FDA-approved and are in use. Additionally, other VLP vaccines are currently under investigation for several families of human viruses, including human immunodeficiency virus, hepatitis virus, rotavirus, parvovirus and influenza virus (Takehara et al., 1988; Conner et al., 1996; Tsao et al., 1996; Quan et al., 2007; Wang et al., 2007; Kang et al., 2009; Klausberger et al., 2014). Several studies have demonstrated the induction of neutralizing antibodies via HTNV VLP immunization using mouse models (Betenbaugh et al., 1995; Li et al., 2010). Importantly, VLP antigens can be processed to present antigens through the major histocompatibility class (MHC) II exogenous pathway and the MHC I endogenous pathway, inducing both CD4^+^ and CD8^+^ T cell-mediated immune responses (Bachmann et al., 1996; Reimann and Schirmbeck, 1999).

Although, VLPs are a promising strategy for HTNV vaccines, developing approaches to enhance the immunogenicity of VLPs is highly desirable. It has been reported that a large variety of active molecules can be attached to the VLP surface (Zdanowicz and Chroboczek, 2016). The present study investigated the hypothesis that immunostimulatory molecules can be incorporated into HTNV VLPs to increase their efficacy.

Granulocyte-macrophage colony-stimulating factor (GM-CSF) is a secreted protein. It could be easily incorporated into HTNV VLPs to form chimeric VLPs (HTNV VLP-GM-CSF) with the help of the membrane-anchored protein glycosylphosphatidylinositol (GPI). Thus, a GPI-anchored form of GM-CSF was expressed in the present study. GM-CSF is known to expand myeloid-derived dendritic cell (DC) populations to augment antigen-induced humoral and cellular immune responses and affect Th1/Th2 cytokine balance. GM-CSF has been extensively used as an effective genetic and protein adjuvant to enhance the immunogenicity of tumor and vaccine antigens (Disis et al., 1996; Kass et al., 2001; Poloso et al., 2002; Skountzou et al., 2007; Chou et al., 2010).

Another immunostimulatory molecule is the CD40 ligand (CD40L), which is a surface molecule and has a membrane-binding region; therefore it could easily be incorporated into HTNV VLPs to form chimeric VLPs (HTNV VLP-CD40L). CD40L is primarily expressed on mature CD4^+^ T cells. The interaction between CD40L and CD40 is important for T cell-dependent B cell activation and isotype switching. The binding of CD40L to CD40 modulates cellular immune responses by inducing the expression of costimulatory molecules that reside on antigen-presenting cells (APCs). Because of the upregulation of costimulatory molecules, APCs are activated, CD4^+^ T cell responses are augmented by increased cytokine production, and CD4^+^-dependent naive CD8^+^ T cells are activated *in vivo* (Skountzou et al., 2007; Lin et al., 2009; Zhang et al., 2010).

In the present study, we incorporated GPI-anchored forms of either GM-CSF or CD40L into HTNV VLPs to produce chimeric VLPs (HTNV VLP-GM-CSF, HTNV VLP-CD40L). The immune responses to these chimeric VLPs and the biological effects of these particles on immune system cells were investigated.

## Materials and methods

### Animals

Adult female C57BL/6 mice (8 weeks old) were purchased from the animal research center of the Fourth Military Medical University (Xi'an, China). The mice were housed in isolated and ventilated cages. All experiments were performed in accordance with protocols approved by the Fourth Military Medical University Medical Ethics Committee (Xi'an, Shaanxi, China; approval no. XJYYLL-2014508). The animals were acclimated to the laboratory environment for 5–7 d before use. While in their home cage environment, the animals were allowed free access to a standard animal diet and tap water. The room was maintained at 20–23°C with a 12 h/12 h light/dark cycle. The animals were deeply anesthetized using inhaled isoflurane (1–3% or as needed) before all operations. All efforts were made to minimize animal suffering, to reduce the number of animals used and utilize alternatives to *in vivo* experiments whenever appropriate or feasible.

### Viruses and cells

HTNV strain 76–118 was provided by our library (Cheng et al., 2014). The pFastBac™ Dual expression system, which includes the pFastBac™ Dual vector, was purchased from Invitrogen (Carlsbad, CA, USA). Sf9 insect cells (ATCC, Rockville, MD, USA), used for packaging and propagating the recombinant baculoviruses (rBVs; Palomares et al., 2012), were maintained in Sf-900 II serum-free medium (SFM; Gibco, Grand Island, NY, USA). Vero E6 cells (ATCC, Rockville, MD, USA) used for the cellular microculture neutralization test and target cell establishment for the CTL assay were maintained in RPMI-1640 medium (Invitrogen, Carlsbad, CA, USA) that was supplemented with 10% fetal bovine serum (FBS; HyClone, Logan, UT, USA).

### Antibodies

Monoclonal antibodies (mAb) 1A8 (specific to the HTNV NP; Xu et al., 2002), Gn-4 (specific to the HTNV Gn), Gc-10 (specific to the HTNV Gc), and 3G1 (with high neutralizing Ab activity against HTNV) were prepared in our laboratory. Our laboratory also supplied the mAb Sp2/0 (Cheng et al., 2014; negative control). Rat anti-mouse GM-CSF and rabbit anti-mouse CD40L antibodies were purchased from Abcam (Cambridge, MA, USA). Other secondary antibodies, including FITC-conjugated antibodies, Cy3-conjugated antibodies, HRP-conjugated antibodies, and PE-conjugated antibodies were purchased from eBioscience (San Diego, CA, USA).

### Protein

The following protein antigens were used for the enzyme-linked immunospot (ELISPOT) assay and enzyme-linked immunosorbent assay (ELISA): (i) purified HTNV GP was obtained from the Biological Product Academy (Lanzhou, Gansu, China), and (ii) HTNV NP was expressed and purified by our laboratory. Soluble recombinant murine GM-CSF and CD40L were purchased from PeproTech (Rocky Hill, NJ, USA) for the *in vitro* cultures.

### Generation of HTNV VLP and chimeric HTNV VLPs

#### DNA constructs

Plasmids containing cDNAs encoding M, S, mouse GPI-GM-CSF, and mouse CD40L were synthesized by TaKaRa (Dalian, Liaoning, China) and then digested with *Eco*RI and *Not*I to obtain DNA fragments of M, S, GM-CSF, and CD40L. Next, these fragments were ligated into pFastBac™ Dual vectors with the polyhedrin (*polh*) promoter. All recombinant DNA constructs were confirmed by restriction enzyme digestion and by DNA sequencing and subsequently transformed into *Escherichia coli* DH10Bac™ cells to isolate recombinant bacmids. All recombinant bacmids were confirmed by PCR amplification using the following primers: pUC/M13 (forward) 5′-CCCAGTCACGACGTTGTAAAACG-3′ and pUC/M13 (reverse) 5′-AGCGGATA ACAATTTCACACAGG-3′. Then, the recombinant bacmids were transfected into Sf9 insect cells using Cellfectin® II Reagent (Invitrogen, Carlsbad, CA, USA) according to the manufacturer's instructions to produce rBVs (rBV-M, rBV-S, rBV-GM-CSF, and rBV-CD40L). The rBV titers were determined using a viral plaque assay according to the manufacturer's instructions. The plaque was used to purify the rBVs.

#### Target protein expression in Sf9 insect cells

The infected Sf9 insect cells were harvested after 48 h, resuspended in 0.01 M PBS (pH 8.0) and prepared as a monolayer on glass slides by acetone fixation to determine the rBV expression (rBV-M, rBV-S, rBV-GM-CSF, and rBV-CD40L). For GP, either mAb Gn-4 or Gc-10 was mixed with rBV-M infected cells and incubated at 4°C for 30 min. As a secondary antibody, FITC-conjugated goat anti-mouse antibody was incubated at 4°C for 30 min. For NP, mAb 1A8 was mixed with rBV-S infected cells and incubated at 4°C for 30 min. As a secondary antibody, FITC-conjugated goat anti-mouse antibody was incubated at 4°C for 30 min. For GM-CSF, a rat anti-mouse GM-CSF antibody was mixed with rBV-GM-CSF infected cells and incubated at 4°C for 30 min. As a secondary antibody, FITC-conjugated goat anti-rat antibody was incubated at 4°C for 30 min. For CD40L, a rabbit anti-mouse CD40L antibody was mixed with rBV-CD40L infected cells and incubated at 4°C for 30 min. As a secondary antibody, FITC-conjugated goat anti-rabbit antibody was incubated at 4°C for 30 min. After staining, the cells were analyzed with fluorescence microscopy using an Olympus IX71 microscope (Tokyo, Japan).

#### Production and purification of HTNV VLP and chimeric HTNV VLPs

Sf9 insect cells were coinfected with rBV-M (10 PFU/cell) and rBV-S (10 PFU/cell) at a multiplicity of infection (MOI) ratio of 1:1 and incubated at 27°C for 72 h to produce HTNV VLPs. HTNV VLP-GM-CSF was produced from Sf9 cells that were coinfected with rBV-M (4 PFU/cell), rBV-S (4 PFU/cell), and rBV-GM-CSF (12 PFU/cell) at an MOI ratio of 1:1:3. HTNV VLP-CD40L was produced from Sf9 cells that were coinfected with rBV-M (4 PFU/cell), rBV-S (4 PFU/cell), and rBV-CD40L (12 PFU/cell) at an MOI ratio of 1:1:3. Three days post infection, the culture supernatants were collected, centrifuged at 1000 rpm for 20 min and filtered through a 0.45 μm pore-size filter. Next, the VLPs were initially concentrated via ultrafiltration using a 15-ml centrifugal filter with a 3 kDa cutoff (Millipore, Billerica, MA, USA) at 3000 rpm for 2 h at 4°C in a Thermo Scientific 75003608 Rotor (Waltham, MA, USA). Then, the VLPs were further purified through a 20–60% discontinuous sucrose gradient at 38,000 rpm for 18 h at 4°C in a Beckman SW 41 Ti Rotor (Fullerton, CA, USA). The VLP bands were collected, washed with PBS, pelleted and resuspended overnight in PBS. The protein concentration of each sample was estimated using a Bio-Rad protein assay (Bio-Rad Laboratories, Hercules, CA, USA) to quantify the purified VLP yield.

### Identification of HTNV VLP and chimeric HTNV VLPs

#### Confocal laser scanning microscope

Sf9 insect cells coinfected with rBV-M, rBV-S, and rBV-GM-CSF or rBV-CD40L were harvested and prepared as a monolayer on glass slides to determine the expression and cellular localization of the proteins that form the components of the VLPs. For GP, mAb Gn-4 was mixed with infected cells and incubated at 4°C for 30 min. As a secondary antibody, FITC-conjugated goat anti-mouse antibody was incubated at 4°C for 30 min. For GM-CSF, a rat anti-mouse GM-CSF antibody was mixed with infected cells and incubated at 4°C for 30 min. As a secondary antibody, Cy3-conjugated goat anti-rat antibody was incubated at 4°C for 30 min. For CD40L, a rabbit anti-mouse CD40L antibody was mixed with infected cells and incubated at 4°C for 30 min. As a secondary antibody, Cy3-conjugated goat anti-rabbit antibody was incubated at 4°C for 30 min. After being stained, the cells were analyzed with a confocal laser-scanning microscope using an Olympus FV1000 microscope (Tokyo, Japan).

#### SDS-PAGE and western-blot

Samples from purified VLPs were lysed in 2× loading buffer (0.08 M Tris [pH 6.8], with 2.0% SDS, 10% glycerol, 0.1 M dithiothreitol, and 0.2% bromophenol blue) and boiled for 5 min for protein analysis. All samples were subjected to sodium dodecyl sulfate-polyacrylamide gel electrophoresis (SDS-PAGE) with identical concentrations (5 or 10 μg). HTNV GP, NP, GM-CSF, and CD40L were probed using mAbs Gn-4/Gc-10, 1A8, rat anti-mouse GM-CSF, and rabbit anti-mouse CD40L antibodies, respectively.

#### Electron microscopy

Sf9 cells coinfected with rBVs were fixed in 0.25% glutaraldehyde and 1% osmium tetraoxide, dehydrated with ethanol, and then embedded in Epon resin to examine VLP production. Thin sections were stained with lead citrate and uranyl acetate and observed using electron microscopy. Samples from purified VLPs were applied to the carbon-coated Formvar grids for 1 min. The grids were then immediately stained with 1% phosphotungstic acid for 1 min and examined using a transmission electron microscope.

#### Immunoelectron microscopy

Purified HTNV VLP-GM-CSF and HTNV VLP-CD40L were detected by immunoelectron microscopy to determine whether the targeted molecules (GM-CSF and CD40L) were efficiently incorporated into the HTNV VLPs. Samples were absorbed onto freshly glow-discharged, carbon- and Formvar-coated, 300-mesh nickel grids for 2 min. The VLPs were stained using anti-GM-CSF and anti-CD40L antibodies, and a secondary antibody labeled with 10 nm colloidal gold. Then, the grids were immediately stained with 1% phosphotungstic acid for 1 min, and examined using a transmission electron microscope.

#### Coimmunoprecipitation

Samples from purified HTNV VLP-GM-CSF and HTNV VLP-CD40L were immunoprecipitated with anti-GM-CSF and anti-CD40L antibodies, respectively, subjected to SDS-PAGE, and probed with mAb Gn-4 to determine whether the targeted molecules (GM-CSF and CD40L) were efficiently incorporated into the HTNV VLPs. The samples from the purified HTNV VLP-GM-CSF and HTNV VLP-CD40L were also immunoprecipitated with Gn-4, subjected to SDS-PAGE, and probed with anti-GM-CSF and anti-CD40L antibodies, respectively.

### Functional characterization of HTNV VLP and chimeric HTNV VLPs

#### Bone marrow cell proliferation

Mouse bone marrow cells were labeled with CFSE (carboxyfluorescein diacetate succinimidyl ester; Molecular Probes, Eugene, OR, USA) at a final concentration of 1 μM. CFSE was quenched by further incubation in serum-containing medium and extensively washed in RPMI medium. The CFSE-labeled bone marrow cells were cultured in RPMI medium in the presence of 20 μg/μl of VLPs to determine the effect of chimeric VLPs on cell proliferation. After incubation at 37°C in 5% CO_2_ for 4 d, the cells were harvested and analyzed by fluorescence-activated cell sorting using a Beckman Epics XL instrument (Fullerton, CA, USA). This experiment was repeated four times.

#### Bone marrow cell differentiation

The bone marrow cultures that were expanded in the presence of VLPs for 4 d were stained with PE-conjugated anti-CD11c and FITC-conjugated anti-CD11b for 1 h at 4°C, fixed in PBS with 1.5% paraformaldehyde, and analyzed using a Beckman Epics XL instrument to evaluate the cell differentiation. This experiment was repeated four times.

#### B cell activation

The mouse spleen cells were cultured in RPMI medium in the presence of 20 μg/μl of VLPs to analyze the phenotypes of the activated cells. After incubation at 37°C in 5% CO_2_ for 4 d, one million cells were stained with PE-conjugated anti-CD69 and FITC-conjugated anti-B220 for 20 min at 4°C. After staining, the cells were washed and fixed in PBS with 1% paraformaldehyde and analyzed using a Beckman Epics XL instrument. This experiment was repeated four times.

### Immunizations

The female C57BL/6 mice were divided into seven groups. Eight mice were assigned to each group (five experimental and two control groups). The first three experimental groups were injected subcutaneously with 200 μl (100 μg) of either purified HTNV VLPs (VLP) or chimeric VLPs (VLP-GM-CSF, VLP-CD40L) per mouse. The other two experimental groups were injected subcutaneously with 200 μl (100 μg) of purified HTNV VLP and 5 μg of soluble recombinant GM-CSF or CD40L per mouse (VLP + GM-CSF, VLP + CD40L). The two control groups were immunized with either 200 μl (100 μg) of commercially purchased inactivated Hantavirus vaccine (Vaccine; Tianyuan, Hangzhou, Zhejiang, China) or with 200 μl of PBS (naive) per mouse. All immunizations were administered three times at 2 week intervals. The mouse (four mice per group) sera were collected individually via retro-orbital plexus puncture 10 d after the last immunization. Additionally, splenocytes were isolated for subsequent assays.

### Characterization of the effect on DCs *in vivo*

#### DC proliferation

Splenocytes from the C57BL/6 mice (four mice per group) were isolated 10 d after the last immunization. The numbers of DCs were counted by gating CD11c and SSC. A total of 100,000 events per sample of splenocytes were collected for each analysis.

#### DC activation

One million splenocytes freshly isolated from the immunized mice (four mice per group) were washed twice with PBS and were stained with PE-conjugated anti-CD86 and FITC-conjugated anti-CD40 for 20 min at 4°C. After staining, the cells were washed and fixed in PBS with 1% paraformaldehyde and analyzed using a Beckman Epics XL instrument. The surface marker expression levels of DCs, CD40, and CD86, were analyzed to evaluate their degree of activation.

#### DC types

Freshly isolated splenocytes collected from the immunized mice (four mice per group) were used to analyze the types of DCs. After washing, the splenocytes were stained with antibodies against surface markers with PerCP/Cy5.5-conjugated anti-CD8a, PE-conjugated anti-CD11b, and FITC-conjugated anti-CD40 for 40 min at 4°C. After staining, the cells were washed and fixed in PBS with 1% paraformaldehyde and analyzed using a Beckman Epics XL instrument. The surface marker expression levels of DCs, CD8a, CD11b, and CD11c were analyzed to determine their type.

### Evaluation of humoral and cellular immune responses

#### Detection of HTNV NP- and GP-specific antibodies

HTNV NP- and GP-specific antibody titers were determined using indirect ELISA. Purified NP and GP were used as coating antigens. Serial dilutions of sera from immunized mice (four mice per group) with an initial dilution of 1:10 were added to the plates and reacted with either NP or GP. The positive controls consisted of 1:100 dilutions of mAbs 1A8 and Gn-4. A 1:1000 dilution of mAb Sp2/0 was used as the negative control. HRP-conjugated goat anti-mouse antibody was used for detection. The absorbance was measured at a wavelength of 450 nm using a Synergy HT ELISA plate reader (Biotec, Dresden, Germany). An absorbance >0.1 and a positive/negative (P/N) value > 2.1 was considered significant. The antibody titers were defined as the reciprocal of the serum dilution with the highest positive response. The geometric mean titer (GMT) was utilized to compare specific antibody titers among all groups.

#### Microneutralization test

The cell microculture neutralization test was performed on monolayers of Vero E6 cells grown in a 96-well tissue culture plate with the HTNV 76–118 strain. Cells that were grown in RPMI-1640 medium supplemented with 10% FBS were plated at a density of 2 × 10^4^ cells per well and cultured for 18–24 h before testing. The sera from the immunized mice (four mice per group) were filtered through 0.22 μm filters, then diluted serially two-fold from an initial 1:10 dilution in RPMI-1640 medium containing 2% FBS, and combined with an equal volume of 100 TCID_50_ HTNV (76–118 strain). After 90 min, the mixture was transferred to monolayers of Vero E6 cells and incubated at 37°C for 7 d in a 5% CO_2_ incubator. Thereafter, the cells were lysed by three consecutive freeze-thaw cycles. HTNV antigens in the cell lysates were detected using sandwich ELISA. The mAb 1A8 was used as a coating antibody, and HRP-conjugated 1A8 was used as the detecting antibody. The mAb 3G1 and Sp2/0 were used as positive and negative controls, respectively. The absorbance was measured at a wavelength of 450 nm using a Synergy HT ELISA plate reader. The neutralizing antibody titer was defined as the maximum dilution of serum that inhibited HTNV infection in 50% of the cells.

#### Detection of cytokines secreted by T cells

The ELISPOT assay was used to determine the frequency of responding T cells that were capable of secreting IFN-γ upon stimulation. The mice were sacrificed 10 d after the final booster immunization. The spleen cells from the immunized mice (four mice per group) were purified in lymphocyte separation medium. CD4^+^ or CD8^+^ T cells from splenocytes were depleted using anti-CD4-coated Dynabeads (Invitrogen Dynal AS, Oslo, Norway) or anti-CD8-coated Dynabeads (Invitrogen Dynal AS, Oslo, Norway). The isolated T cells (1 × 10^6^ cells in 100 μl) were added to each well of pre-coated IFN-γ microtiter plates (Mabtech, Stockholm, Sweden), and stimulated with a mixture of HTNV purified GP and NP (10 μg/ml) or with the positive mitogenic stimulator concanavalin A (ConA, 4 μg/ml). The isolated T cells incubated with 100 μl of 2% FBS supplemented RPMI-1640 medium were used as negative or background controls. These plates were incubated at 37°C for 18 h. Cytokine ELISPOT development was performed according to the manufacturer's instructions. The spots were counted using an ELISPOT reader system (AID, Strasberg, Germany). The results were expressed as the mean number of specific IFN-γ spot-forming cells per 1 × 10^6^ splenocytes. The assays for IL-2, IL-4, and IL-10 secretion were similar to those described for IFN-γ.

#### Cytotoxicity assay

A CytoTox 96™ nonradioactive cytotoxicity assay kit (Promega, Madison, WI, USA) was used according to the manufacturer's instructions to detect the level of specific toxicity in response to the Vero E6 cells (target cells) infected with the HTNV 76–118 strain. The target cells were plated onto 96-well U-bottomed microtiter plates at 1 × 10^4^ cells per well and a volume of 50 μl. The splenocytes from the immunized mice (four mice per group; effector cells) were prepared as described above and added to a final volume of 50 μl with effector/target (E/T) ratios of 100:1, 50:1, and 20:1. Normal splenocytes were added as a negative control. The assay plate included the following cells as controls: spontaneous lactate dehydrogenase (LDH) release from effector cells (50 μl of target cells and 50 μl of 5% FBS RPMI-1640 medium), spontaneous LDH release from target cells (50 μl of target cells and 50 μl of 5% FBS RPMI-1640 medium), maximum LDH release from target cells (50 μl of target cells, 50 μl of 5% FBS RPMI-1640 medium, and 10 μl of the lysis solution), a volume correction control (100 μl of 5% FBS RPMI-1640 medium and 10 μl of the lysis solution), and a culture medium background control (100 μl of 5% FBS RPMI-1640 medium). The percentage of Vero E6 cell lysis was calculated according to the following formula: % cytotoxicity = [(*E* − *S*_*t*_ − *S*_*e*_)/(*M* − *S*_*t*_)] × 100 (E, LDH release from effector-target co-culture cells; St, spontaneous LDH release from target cells; Se, spontaneous LDH release from effector cells; M, maximum LDH release from target cells).

### Animal protection experiments

Ten days after the final booster immunization, the C57BL/6 mice (four mice per group) were infected with the HTNV 76–118 strain (1 × 10^5^ pfu/mouse) by intramuscular injection. Then, the mice were killed after 3 d, and the major tissues, including the cerebrum, heart, liver, spleen, lungs, and kidneys, were collected individually for subsequent assays.

#### Detection of HTNV antigens in the tissues by ELISA

The tissue samples from the HTNV-infected mice (four mice per group) were weighed, diluted in PBS, and then freeze-thawed (−80/37°C) three times after being ground to prepare 10% (g/ml) tissue suspensions. The samples were centrifuged at 12,000 rpm for 30 min at 4°C, and the supernatants were collected. The HTNV antigens in the supernatants were detected using sandwich ELISA. The mAb 1A8 was used as a coating antibody, and HRP-conjugated 1A8 was used as the detecting antibody. The normal tissue supernatants were used as negative controls. The absorbance was measured at a wavelength of 450 nm using a Synergy HT ELISA plate reader. An OD450 value exceeding 2.1-fold of the negative controls was considered a positive result.

#### Detection of HTNV nucleic acids in tissues by qRT-PCR

The total cellular RNAs in the tissue samples from the HTNV-infected mice (four mice per group) were extracted using an RNAprep Pure Tissue Kit (Tiangen, Beijing, China). Then, 1 μg of total cellular RNA from each sample was amplified using a SYBR Premix Ex Taq II Kit and the following HTNV RNA-specific primers: HTNV (forward) 5′-GATCAGTCACAGTCTAGTCA-3′ and HTNV (reverse) 5′-TGATTCTTCCACCATTTTGT-3′. GAPDH was included in the qRT-PCR as an internal control for cellular RNA using the following mouse GAPDH-specific primers: GAPDH (forward) 5′-AGGCCGGTGCTGAGTATG TC-3′ and GAPDH (reverse) 5′-TGCCTGCTTCACCACCTTCT-3′.

### Statistical analysis

All data were expressed as the mean ± the standard error of mean (SEM) and are representative of at least two independent experiments. The Student's *t*-test was used to determine significant differences between the paired experimental and control groups. One-way ANOVA was used to determine statistically significant differences among the experimental groups. A *p*-value of 0.05 or less was considered significant.

## Results

### HTNV VLP and chimeric HTNV VLPs were produced and purified successfully

**Figure 1:**
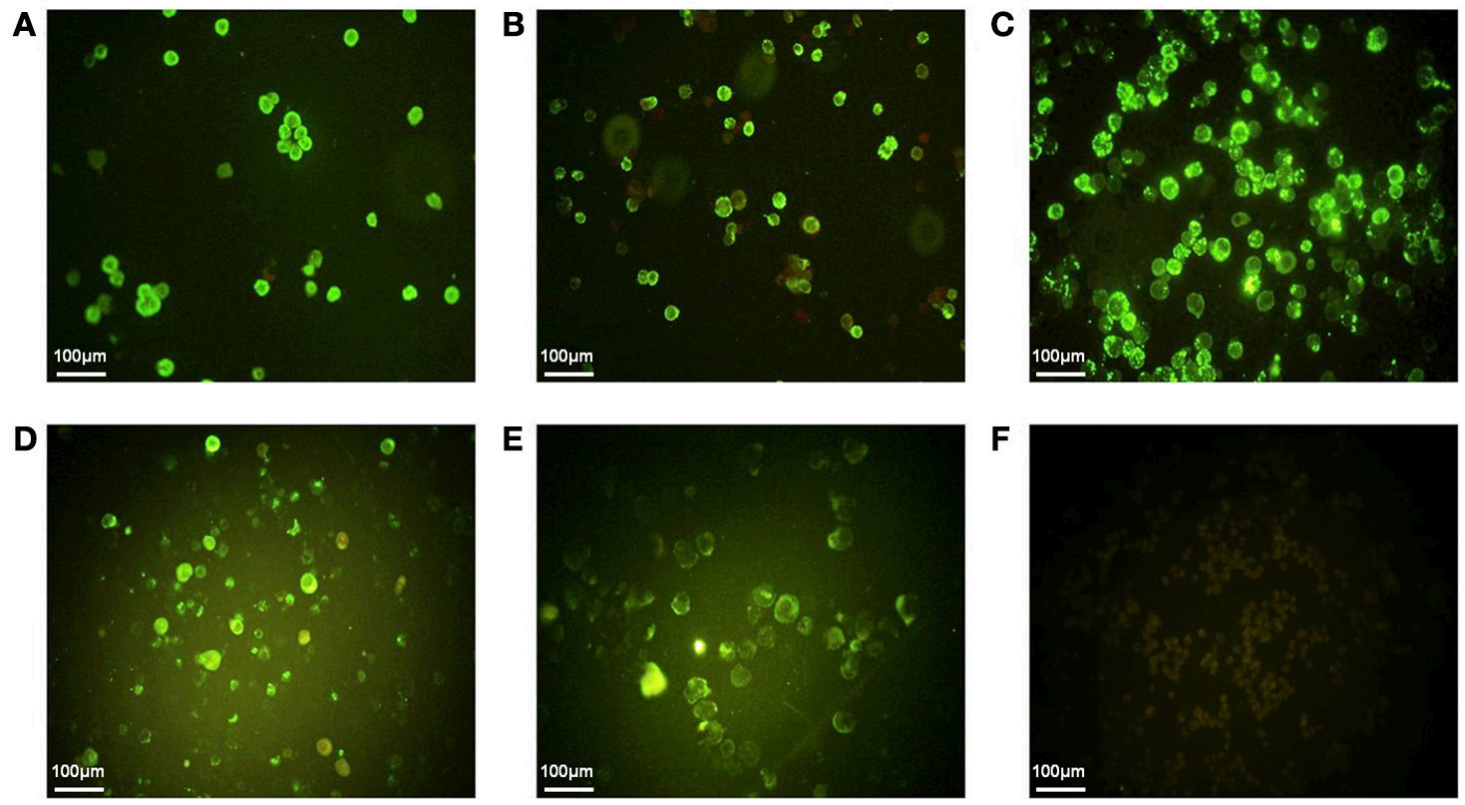
**The IFA of GP, NP, GM-CSF, and CD40L expression in Sf9 insect cells. (A)** HTNV GP expression was detected using mAb Gn-4 (Gn-specific) in Sf9 insect cells infected with rBV-M. **(B)** HTNV GP expression was detected using mAb Gc-10 (Gc-specific) in Sf9 insect cells infected with rBV-M. **(C)** HTNV NP expression was detected using mAb 1A8 (NP-specific) in Sf9 insect cells infected with rBV-S. **(D)** GM-CSF expression was detected using rat anti-mouse GM-CSF antibody in Sf9 insect cells infected with rBV-GM-CSF. **(E)** CD40L expression was detected using rabbit anti-mouse CD40L antibody in Sf9 insect cells infected with rBV-CD40L. **(F)** Normal Sf9 insect cells were detected using a mixture of the above-mentioned monoclonal antibodies (200×).

To express HTNV GP and NP, GM-CSF and CD40L in Sf9 insect cells, first, we cloned these genes into baculovirus shuttle vectors and produced rBVs. Then, we confirmed the expression of GP, NP, GM-CSF, and CD40L by infecting Sf9 insect cells with the rBVs and by analyzing these cells using an immunofluorescence assay (IFA). All the target proteins were expressed in Sf9 insect cells (Figure 1). HTNV VLPs were produced by Sf9 insect cells coinfected with rBV-M and rBV-S. HTNV VLP-GM-CSF was produced by Sf9 insect cells coinfected with rBV-M, rBV-S, and rBV-GM-CSF and HTNV VLP-CD40L was produced by Sf9 insect cells coinfected with rBV-M, rBV-S, and rBV-CD40L. Then, the VLPs were harvested from the culture supernatants and purified using sucrose gradient ultracentrifugation. The BCA Protein Assay Kit was used to measure the VLP concentration after purification via sucrose gradient ultracentrifugation. The concentration of HTNV VLPs, VLP-CD40L, and VLP- GM-CSF reached up to 3.648, 3.824, and 3.301 mg/ml, respectively.

**Figure 2:**
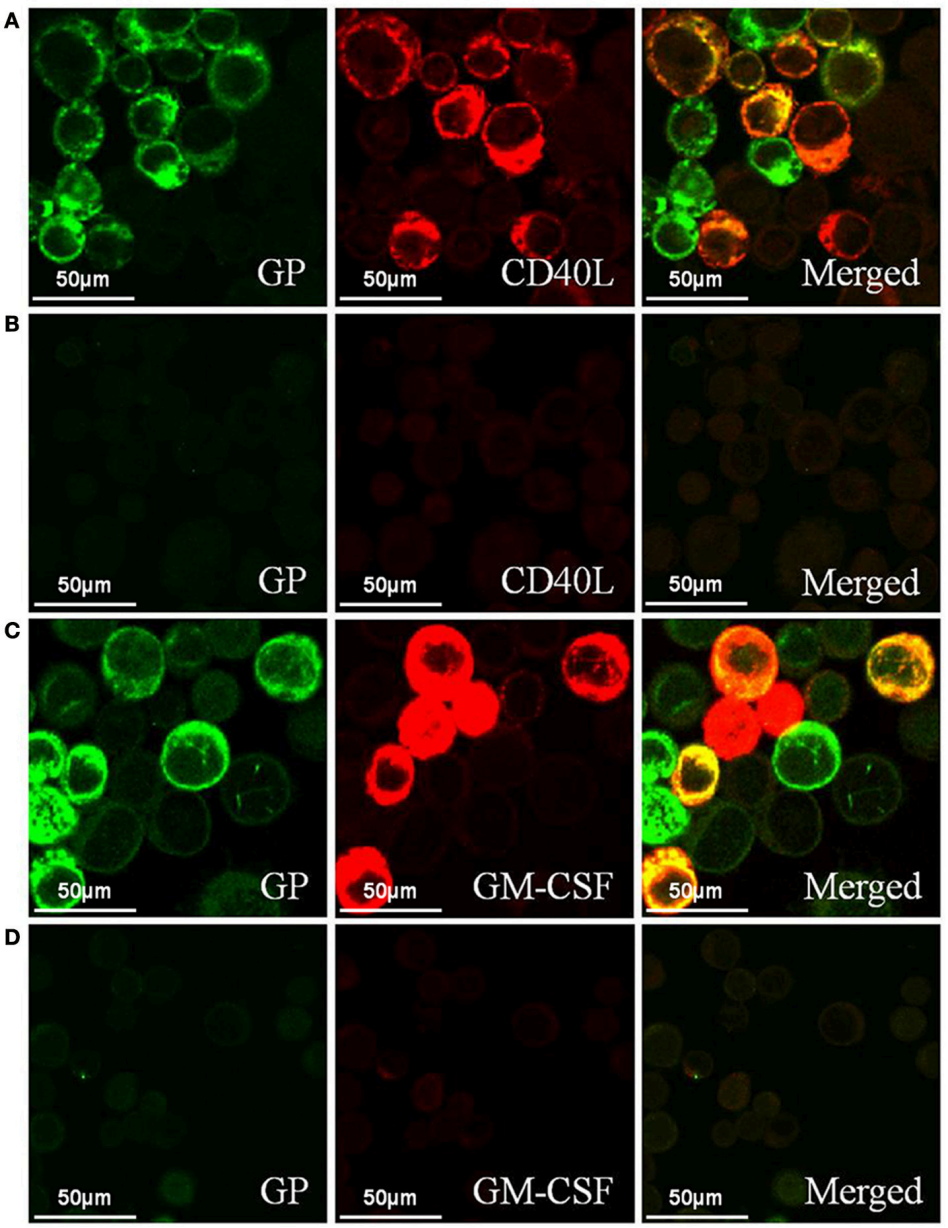
**Confocal laser scanning images of the Sf9 insect cells coinfected with rBVs. (A)** Sf9 insect cells coinfected with rBV-M, rBV-S, and rBV-CD40L. **(B)** Normal Sf9 insect cells. **(C)** Sf9 insect cells coinfected with rBV-M, rBV-S, and rBV-GM-CSF. **(D)** Normal Sf9 insect cells (600 ×). HTNV GP expression was detected using mAb Gn-4 (Gn-specific) and the FITC-conjugated goat anti-mouse antibody was used as a secondary antibody. CD40L expression was detected using a rabbit anti-mouse CD40L antibody and the Cy3-conjugated goat anti-rabbit antibody was used as a secondary antibody. GM-CSF expression was detected using a rat anti-mouse GM-CSF antibody and the Cy3-conjugated goat anti-rat antibody was used as a secondary antibody.

**Figure 3:**
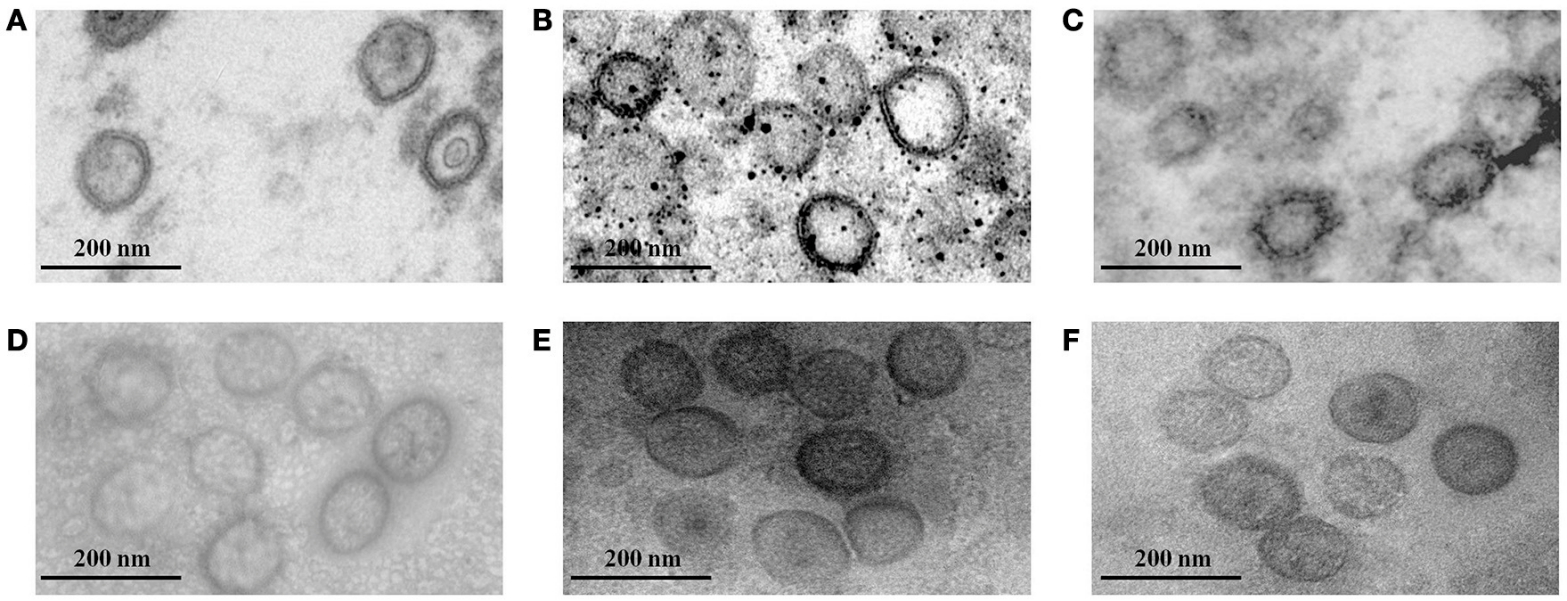
**Electron microscopy of VLPs. (A)** Electron microscopy of the HTNV VLPs in Sf9 insect cells coinfected with rBV-M and rBV-S. **(B)** Electron microscopy of HTNV VLP-GM-CSF in Sf9 insect cells coinfected with rBV-M, rBV-S, and rBV-GM-CSF. **(C)** Electron microscopy of HTNV VLP-CD40L in Sf9 insect cells coinfected with rBV-M, rBV-S, and rBV-CD40L. **(D)** Electron microscopy of purified HTNV VLPs. **(E)** Electron microscopy of purified HTNV VLP-GM-CSF. **(F)** Electron microscopy of purified HTNV VLP-CD40L (80,000×).

**Figure 4:**
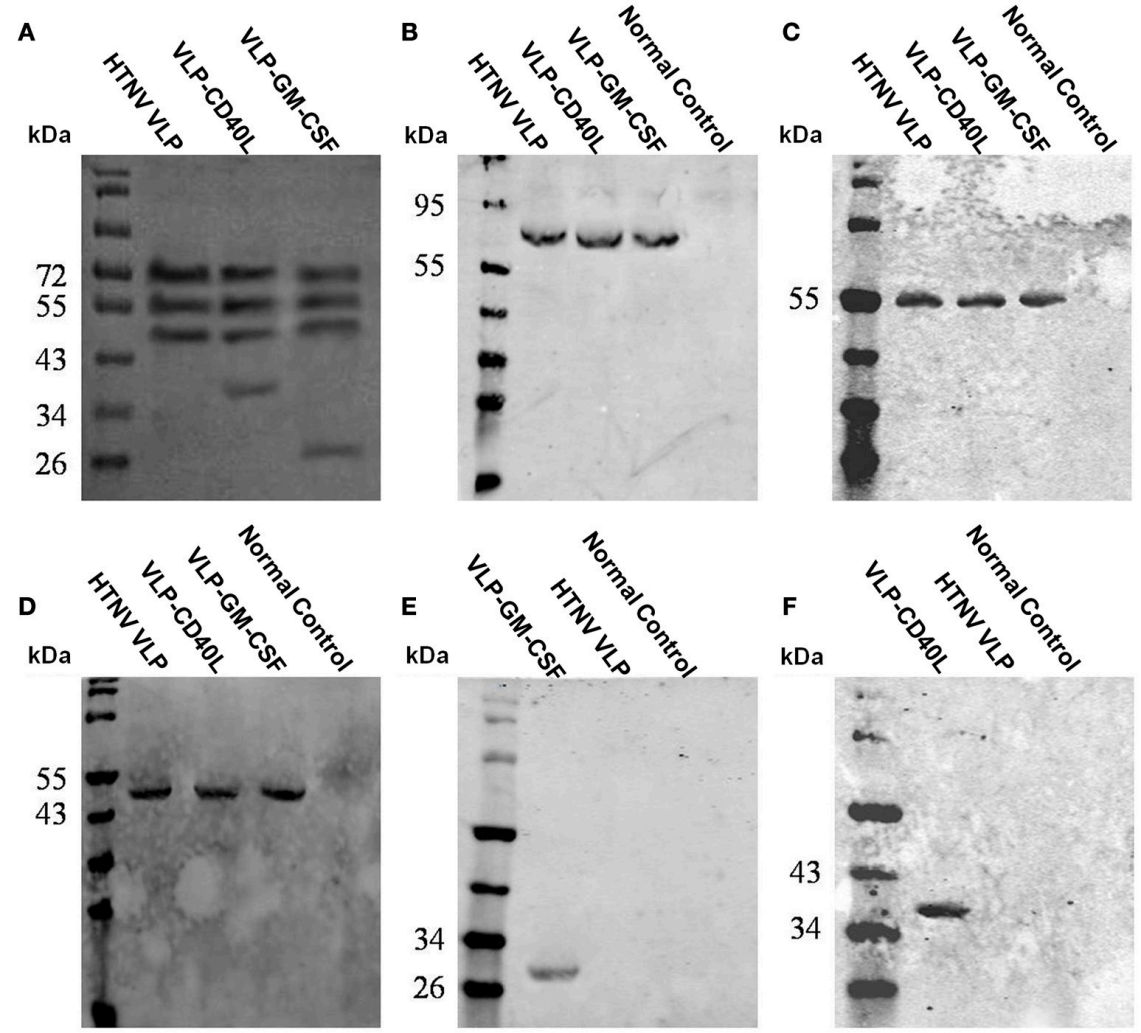
**Characterization of purified VLPs using SDS-PAGE and Western blot. (A)** SDS-PAGE of the purified VLPs. **(B)** Western blot analysis of the purified VLPs using Gn-4. **(C)** Western blot analysis of the purified VLPs using Gc-10. **(D)** Western blot analysis of the purified VLPs using 1A8. **(E)** Western blot analysis of the purified VLPs using rat anti-mouse GM-CSF. **(F)** Western blot analysis of purified VLPs using rabbit anti-mouse CD40L.

We determined the expression and cellular localization of the proteins forming the VLP components in the Sf9 insect cells. We could detect the expression of HTNV GP and CD40L in the same Sf9 insect cells coinfected with rBV-M, rBV-S, and rBV-CD40L (Figure 2A). Similarly, we could detect the expression of HTNV GP and GM-CSF in the same Sf9 insect cells coinfected with rBV-M, rBV-S, and rBV-GM-CSF (Figure 2C). Normal Sf9 insect cells were used as negative control (Figures 2B,D). We also collected cells coinfected with rBVs to detect VLP production using electron microscopy; the particle sizes ranged from ~100 nm in diameter (Figures 3A–C). The purified VLPs were routinely tested for integrity and homogeneity using electron microscopy; the particle sizes ranged from ~100 nm in diameter (Figures 3D–F). The VLPs were also analyzed using SDS-PAGE and Western blot. The results of the SDS-PAGE analysis indicated that all the VLPs exhibited three Coomassie-stained bands at apparent molecular weights of ~72, 55, and 50 kDa and that HTNV VLP-GM-CSF and HTNV VLP-CD40L also showed a 29 and 37 kDa band (Figure 4A), which were similar to the molecular weights of Gn, Gc, NP, GM-CSF, and CD40L, respectively. Western blot analysis indicated that Gn, Gc, and NP from all the purified VLPs were detected by mAbs Gn-4, Gc-10, and 1A8, respectively (Figures 4B–D). This analysis also showed that GM-CSF and CD40L from the purified HTNV VLP-GM-CSF and HTNV VLP-CD40L were detected by rat anti-mouse GM-CSF and by rabbit anti-mouse CD40L antibodies, respectively (Figures 4E,F).

**Figure 5:**
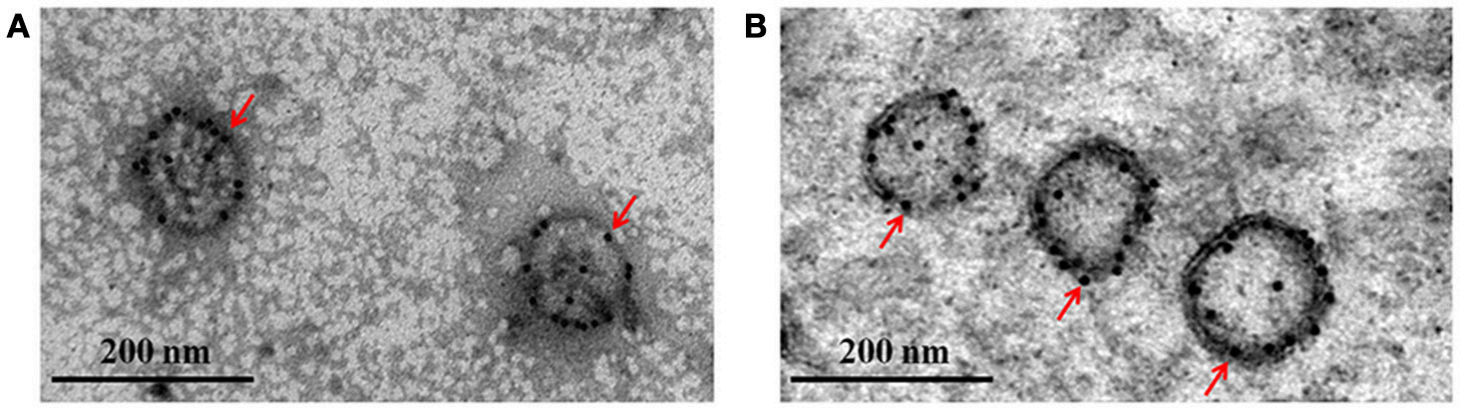
**Characterization of the purified VLPs using immunoelectron microscopy**. Purified HTNV VLP-GM-CSF and HTNV VLP-CD40L were stained using anti-GM-CSF or anti-CD40L antibodies, respectively, and a secondary antibody labeled with 10 nm colloidal gold. **(A)** Immunoelectron microscopy of purified HTNV VLP-GM-CSF. **(B)** Immunoelectron microscopy of purified HTNV VLP-CD40L (80,000×).

**Figure 6:**
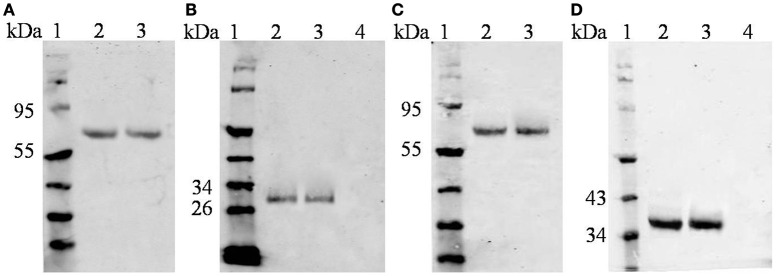
**Characterization of purified VLPs using coimmunoprecipitation. (A)** HTNV VLP-GM-CSF was immunoprecipitated with anti-GM-CSF antibody and then probed with anti-GP antibody (Gn-4). **(B)** HTNV VLP-GM-CSF was immunoprecipitated with anti-GP antibody (Gn-4) and then probed with anti-GM-CSF antibody. (**A,B** Lanes: 1, protein marker; 2, HTNV VLP-GM-CSF; 3, HTNV VLP-GM-CSF as a positive control without precipitation; and 4, HTNV VLPs as a negative control) **(C)** HTNV VLP-CD40L was immunoprecipitated with anti-CD40L antibody and then probed with anti-GP antibody (Gn-4). **(D)** HTNV VLP-CD40L was immunoprecipitated with anti-GP antibody (Gn-4) and then probed with anti-CD40L antibody. (**C,D** Lanes: 1, protein marker; 2, HTNV VLP-CD40L; 3, HTNV VLP-CD40L as a positive control without precipitation; and 4, HTNV VLPs as a negative control).

We used immunoelectron microscopy to determine whether GM-CSF and CD40L were directly incorporated into the HTNV VLPs. The particle sizes were ~100 nm in diameter, and the colloidal gold could be detected around the particles (Figures 5A,B). The immunoelectron microscopy results confirmed that the GM-CSF and CD40L were directly incorporated into the HTNV VLPs. We also used a coimmunoprecipitation assay to determine whether GM-CSF and CD40L were directly incorporated into the HTNV VLPs. HTNV VLP-GM-CSF was immunoprecipitated with rat anti-mouse GM-CSF antibody, and the proteins were probed with Gn-4 mAb after separation by SDS-PAGE. HTNV GP coprecipitated, indicating that GM-CSF and HTNV GP were present in the same VLP structures (Figure 6A). Similarly, GM-CSF coprecipitated when HTNV VLP-GM-CSF was first immunoprecipitated with the anti-GP antibody (Gn-4) and the blots were subsequently probed with rat anti-mouse GM-CSF (Figure 6B). Analogous experiments confirmed the incorporation of CD40L into the HTNV VLPs (Figures 6C,D).

Taken together, these results demonstrate that HTNV VLPs and chimeric HTNV VLPs were produced and purified successfully.

### GM-CSF and CD40L incorporated into HTNV VLPs maintained their biological activities

**Figure 7:**
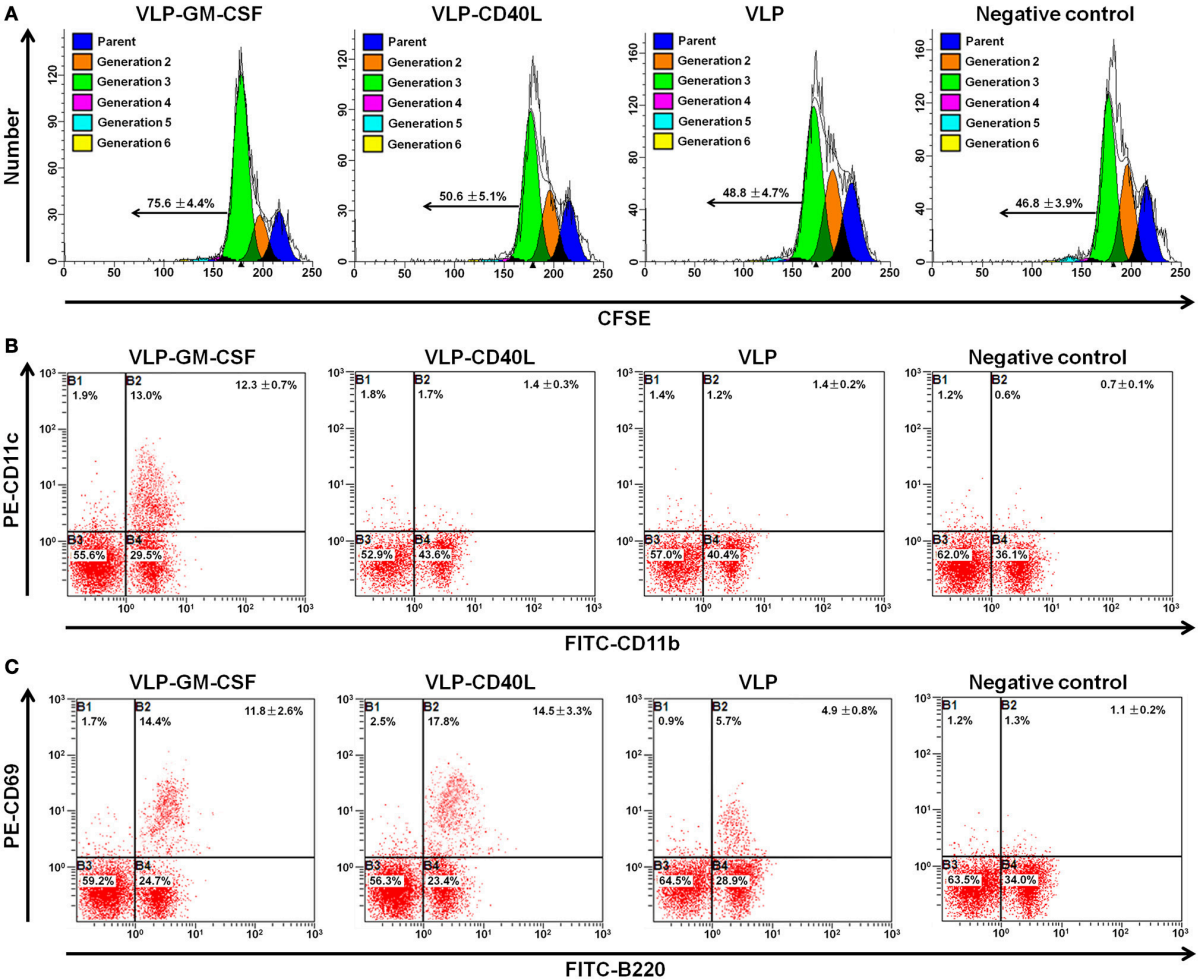
**Flow cytometry characterization of the biological activities of GM-CSF and CD40L incorporated into HTNV VLPs**. All the experiments were repeated four times. **(A)** Single-cell suspensions of bone marrow cells from C57BL/6 mice were labeled with 1 μM CFSE and cultured *in vitro* in the presence of 20 μg/ml of VLPs. Four days later, the cells were harvested, and the extent of cellular proliferation was determined using CFSE dilution. Representative flow cytometric plots are shown. The numbers indicate the percentages of generation 3 (average ± standard error). **(B)** Single-cell suspensions of bone marrow cells from C57BL/6 mice were cultured *in vitro* in the presence of 20 μg/ml of VLPs. Four days later, the cells were stained with FITC-conjugated anti-CD11b and PE-conjugated anti-CD11c antibodies. Representative flow cytometric plots are shown. The numbers indicate the percentages of gated populations in the double-positive quadrant (average ± standard error). **(C)** *In vitro* cultures were set up as described for **(B)**, and the cells were stained with FITC-conjugated anti-B220 and PE-conjugated anti-CD69 antibodies. Representative flow cytometric plots are shown. The numbers indicate the percentages of gated populations in the double-positive quadrant (average ± standard error).

GM-CSF is a potent activator that induces the proliferation and differentiation of bone marrow cells. GM-CSF and CD40L also induce the activation of splenic B cells. First, we tested whether these VLPs could induce the proliferation of bone marrow cells to determine whether GM-CSF and CD40L incorporated into HTNV VLPs maintained their endogenous activities. We labeled bone marrow cells with 1 μM CFSE and incubated the cells for 4 d in the presence of 20 μg/ml of VLPs. As expected, we found that HTNV VLP-GM-CSF effectively increased the percentage of generation 3 bone marrow cells compared to the negative control (*p* = 0.003). We also found that HTNV VLP-GM-CSF induced the proliferation of bone marrow cells compared to the HTNV VLPs (*p* = 0.014). In contrast, HTNV VLP-CD40L did not induce significant bone marrow cell proliferation compared to the HTNV VLPs (*p* = 0.245 for HTNV VLPs and *p* = 0.412 for the negative control; Figure 7A).

Then, we analyzed the differentiation of bone marrow cells using flow cytometry to determine the presence of DCs. We observed that bone marrow cells that were cultured in the presence of HTNV VLP-GM-CSF contained significantly higher numbers of CD11c^+^ CD11b^+^ myeloid DCs compared to the HTNV VLPs and the negative control (*p* = 0.006 for HTNV VLP, *p* = 0.001 for negative control). CD40L incorporated into the VLPs had no effect on the stimulation of bone marrow cell differentiation compared to the HTNV VLPs (*p* = 0.312; Figure 7B).

Both GM-CSF and CD40L play critical roles in B cell activation. To determine whether GM-CSF and CD40L incorporated into HTNV VLPs were capable of activating B cells, we cultured splenocytes for 4 d in the presence of 20 μg/ml of VLPs. Then, we analyzed B cell activation by examining the expression of the activation markers CD69 and B220 using flow cytometry. We found that the numbers of activated CD69^+^ B220^+^ cells increased when splenocytes were cultured with VLPs relative to the negative control (*p* = 0.006 for VLP-GM-CSF, *p* = 0.003 for VLP-CD40L, and *p* = 0.012 for HTNV VLPs). We also found that HTNV VLP-GM-CSF and HTNV VLP-CD40L increased the number of activated CD69^+^ B220^+^ cells compared to HTNV VLPs (*p* = 0.014 for VLP-GM-CSF, *p* = 0.007 for VLP-CD40L; Figure 7C).

Together, these results demonstrated that GM-CSF and CD40L incorporated into HTNV VLPs maintained their biological activities.

### GM-CSF and CD40L incorporated into HTNV VLPs induced proliferation, activation, and switching of DCs *in vivo*

**Figure 8:**
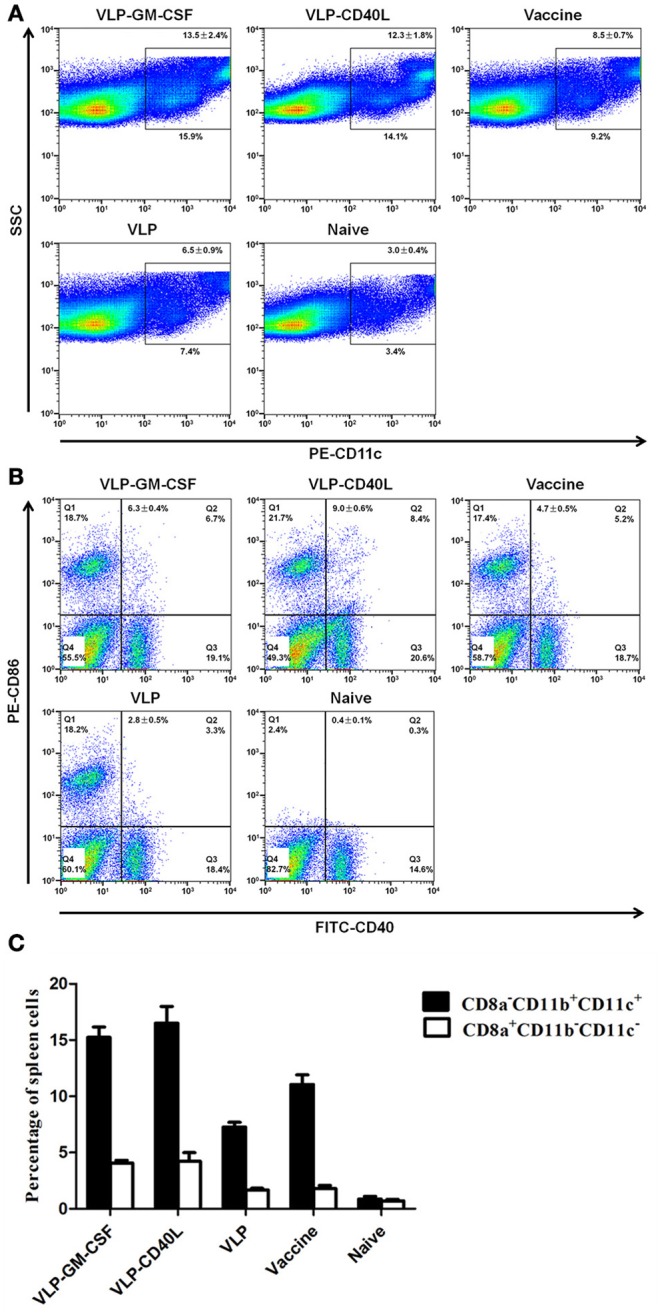
**Flow cytometric characterization of the proliferation, activation and DC switch in the spleens of immunized mice (four mice per group)**. **(A)** Single-cell suspensions of spleens from immunized mice were gated with SSC and PE-conjugated anti-CD11c antibodies. Representative flow cytometric plots are shown. The numbers indicate the percentages of DCs (average ± standard error). **(B)** Single-cell suspensions of spleens from immunized mice were stained with FITC-conjugated anti-CD40 and PE-conjugated anti-CD86 antibodies. Representative flow cytometric plots are shown. The numbers indicate the percentages of gated populations in the double-positive quadrant (average ± standard error). **(C)** Single-cell suspensions of spleens from immunized mice were stained with APC-conjugated anti-CD8a, FITC-conjugated anti-CD11b, and PE-conjugated anti-CD11c antibodies. The figure shows the percentage of DC1 (CD8a-CD11b+CD11c+) and DC2 (CD8a+CD11b− CD11c−) cells in the spleens of immunized mice. The data are expressed as the average ± standard error.

The numbers of DCs from the splenocytes of immunized mice were counted by gating CD11c and SSC. As expected, we found that both HTNV VLP-GM-CSF and HTNV VLP-CD40L effectively increased the number of DCs compared to the negative control (*p* = 0.013 for VLP-GM-CSF, *p* = 0.009 for VLP-CD40L) and HTNV VLPs (*p* = 0.021 for VLP-GM-CSF, *p* = 0.011 for VLP-CD40L). We also found that HTNV VLP-GM-CSF and HTNV VLP-CD40L increased the number of DCs compared to the vaccine (*p* = 0.037 for VLP-GM-CSF, *p* = 0.014 for VLP-CD40L; Figure 8A).

To determine whether GM-CSF and CD40L incorporated into HTNV VLPs were capable of activating DCs in the spleen *in vivo*, we assessed DC activation by examining the expression of the activation markers CD86 and CD40 using flow cytometry. We found that the number of activated DCs from the chimeric HTNV VLPs increased compared to the negative control (*p* = 0.009 for VLP-GM-CSF, *p* = 0.003 for VLP-CD40L) and HTNV VLPs (*p* = 0.012 for VLP-GM-CSF, *p* = 0.007 for VLP-CD40L). We also found that HTNV VLP-GM-CSF and HTNV VLP-CD40L increased the number of activated DCs compared to the vaccine (*p* = 0.034 for VLP-GM-CSF, *p* = 0.017 for VLP-CD40L; Figure 8B).

Because the DC1 population expressed CD8a-CD11b+CD11c+ as their specific surface markers, and the DC2 population expressed CD8a+CD11b-CD11c- as their specific surface markers, we analyzed the switching of DC by examining the expression of the CD8a, CD11b and CD11c+ markers using flow cytometry. The number of DC1 cells (CD8a-CD11b+ CD11c+) was greater than the number of DC2 cells in each group (except the negative control). We also found that HTNV VLP-GM-CSF and HTNV VLP-CD40L could effectively increase the numbers of DC1 cells compared to HTNV VLPs (*p* = 0.006 for VLP-GM-CSF, *p* = 0.003 for VLP-CD40L; Figure 8C).

### CD40L and GM-CSF incorporated into HTNV VLPs elicited enhanced humoral immune responses

**Figure 9:**
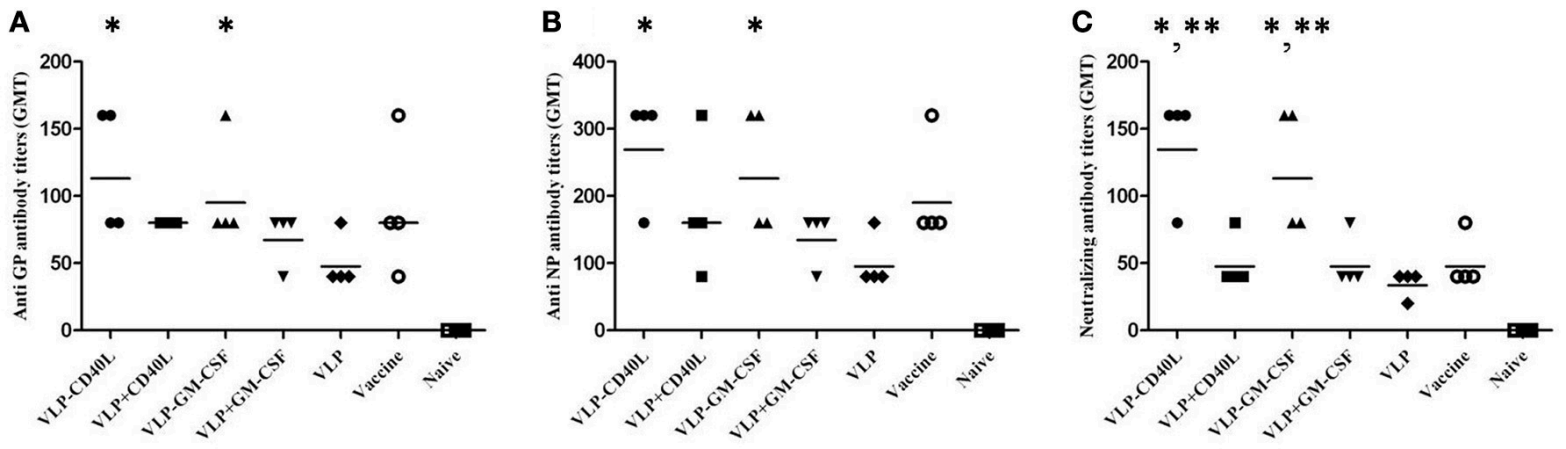
**Characterization of humoral immune responses elicited by HTNV VLPs incorporated with either GM-CSF or CD40L**. The mouse sera were collected as described in the Section Materials and Methods. The results were expressed as the mean value ± SD of four independent experiments. The horizontal lines denote the GMT values. VLP-GM-CSF and VLP-CD40L indicate the HTNV chimeric VLPs, VLP+GM-CSF, and VLP+CD40L indicate the HTNV VLPs with either soluble GM-CSF or CD40L. **(A)** Anti-GP antibody titers were detected using purified GP. **(B)** Anti-NP antibody titers were detected using purified NP. **(C)** Neutralizing antibody titers were detected by determining the ability of the sera to neutralize the live HTNV 76–118 strain. ^*^p < 0.05 compared to the HTNV VLP group; ^**^p < 0.05 compared to the inactivated hantavirus vaccine group.

The immunized mouse sera were collected individually and used to detect the titers of GP- or NP-specific antibodies using ELISA to determine whether HTNV VLP-CD40L and HTNV VLP-GM-CSF could elicit specific and enhanced immune responses against the HTNV major antigens GP and NP. The GMT of the mice immunized with the HTNV VLP against GP and NP were 47.6 and 95.1, respectively; however, the GMT of mice immunized with the HTNV VLP incorporated with CD40L or GM-CSF against GP and NP were 113.1 and 269.1 for CD40L and 95.1 and 226.3 for GM-CSF, respectively. The results demonstrated that the HTNV VLP could enhance immune responses against the HTNV major antigens GP (*p* = 0.03 for VLP-CD40L and *p* = 0.031 for VLP-GM-CSF) and NP (*p* = 0.007 for VLP-CD40L and *p* = 0.03 for VLP-GM-CSF) after being incorporated with CD40L and GM-CSF. We also found that the GMTs of HTNV VLP-CD40L and HTNV VLP-GM-CSF were not significantly higher than those of the inactivated hantavirus vaccine group against GP (80) (*p* = 0.41 for VLP-CD40L and *p* = 0.77 for VLP-GM-CSF) and NP (190.3) (*p* = 0.21 for VLP-CD40L and *p* = 0.54 for VLP-GM-CSF), although the lowest titers of the HTNV VLP-CD40L and HTNV VLP-GM-CSF groups against GP were both 80, which is higher than that of the inactivated hantavirus vaccine group (40). The GMTs of mice immunized with the HTNV VLP with added soluble CD40L or GM-CSF against GP and NP were 80 and 160 for soluble CD40L and 67.3 and 134.5 for soluble GM-CSF, respectively. The GMTs of the mice immunized with PBS against GP and NP were extremely low (GMT < 10; Figures 9A,B).

Next, we assessed the neutralizing activity of the induced antibodies by determining the ability of the serum to neutralize the live HTNV strain 76–118. The GMT of the neutralizing antibody from mice immunized with the HTNV VLPs was 33.6; however, the GMT of the neutralizing antibody from mice immunized with the HTNV VLPs incorporated with CD40L and GM-CSF were 134.5 and 113.1, respectively. These results demonstrated that the HTNV VLP could elicit enhanced neutralizing activity after incorporation with CD40L and GM-CSF (*p* = 0.002 for VLP-CD40L and *p* = 0.011 for VLP-GM-CSF). We also found that the GMT of the neutralizing antibody from mice immunized with HTNV VLP-CD40L and HTNV VLP-GM-CSF were significantly higher than the GMT of the inactivated Hantavirus vaccine (47.6) (*p* = 0.007 for VLP-CD40L and *p* = 0.032 for VLP-GM-CSF). The GMTs of the neutralizing antibody from mice immunized with HTNV VLPs with added soluble CD40L or GM-CSF were both 47.6, which was similar to the inactivated hantavirus vaccine group. The GMT of the neutralizing antibody from mice immunized with PBS was extremely low (GMT < 10; Figure 9C).

Based on these results, we conclude that CD40L and GM-CSF incorporated into HTNV VLPs elicited enhanced humoral immune responses.

### CD40L and GM-CSF incorporated into HTNV VLPs elicited enhanced cellular immune responses

**Figure 10:**
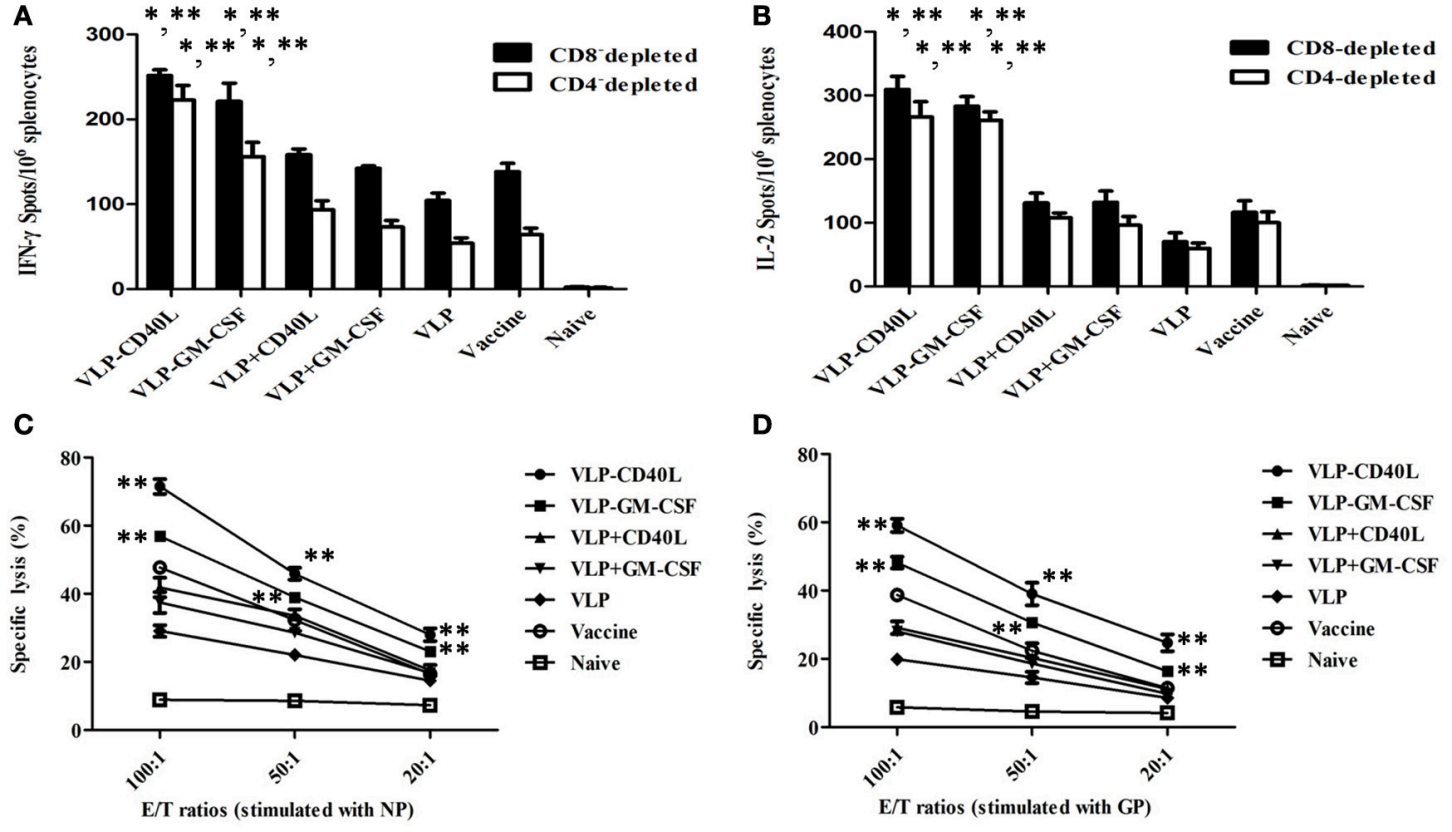
**Characterization of cellular immune responses elicited by HTNV VLPs incorporated with either GM-CSF or CD40L**. The mouse splenocytes and the CD4 or CD8-depleted splenocytes were collected as described in the Section Materials and Methods. The results are expressed as the mean value ± SD of four independent experiments. VLP-GM-CSF or VLP-CD40L indicates the HTNV chimeric VLPs, VLP+GM-CSF, or VLP+CD40L indicates the HTNV VLPs with soluble GM-CSF or CD40L. **(A)** ELISPOT analysis of IFN-γ secreted by CD4 or CD8-depleted splenocytes. **(B)** ELISPOT analysis of IL-2 secreted by CD4 or CD8-depleted splenocytes. **(C)** Cytotoxicity assay of splenocytes stimulated with NP. **(D)** Cytotoxicity assay of splenocytes stimulated with GP. ^*^p < 0.05 compared to the HTNV VLP group; ^**^p < 0.05 compared to the inactivated hantavirus vaccine group.

First, we measured cytokine production using ELISPOT assays as an indicator of cellular immune responses to compare T cell responses to VLPs. Briefly, CD8-depleted splenocytes from HTNV VLP-CD40L and HTNV VLP-GM-CSF immunized mice stimulated with a mixture of purified HTNV NP and GP exhibited a significantly higher number of IFN-γ spots compared to the HTNV VLP group (*p* = 0.007 for VLP-CD40L and *p* = 0.012 for VLP-GM-CSF). The highest numbers were observed in the HTNV VLP-CD40L group. We also found that the numbers of IFN-γ spots from the HTNV VLP-CD40L and HTNV VLP-GM-CSF groups were higher than those of the inactivated Hantavirus vaccine group (*p* = 0.014 for VLP-CD40L and *p* = 0.017 for VLP-GM-CSF). The number of IFN-γ spots in the HTNV VLP group with added soluble CD40L or GM-CSF was not significantly higher than the inactivated Hantavirus vaccine group. The number of IFN-γ spots in mice immunized with PBS was extremely low. The results were similar in the CD4-depleted splenocytes (Figure 10A). The changes in the IL-2 levels were similar to those observed for IFN-γ (Figure 10B). The IL-4 and IL-10 levels did not change significantly in any of the immunization groups (data not shown). Based on these results, CD40L and GM-CSF incorporated into HTNV VLPs induced effective IFN-γ and IL-2 responses.

LDH release from HTNV-infected Vero E6 cells targeted by vaccination-activated splenocytes was measured using a Cytotox 96 non-radioactive cytotoxicity assay kit. The cytotoxicity of splenocytes from mice immunized with VLPs was enhanced in accordance with the E/T ratio, which was the most significant at the 100:1 ratio. Among the experimental groups, splenocytes from mice immunized with either HTNV VLP-CD40L or with HTNV VLP-GM-CSF and then stimulated with purified HTNV NP showed higher specific cytotoxic activity compared to the HTNV VLP group with E/T ratios of 100:1 (*p* = 0.0009 for VLP-CD40L and *p* = 0.0003 for VLP-GM-CSF), 50:1 (*p* = 0.0003 for VLP-CD40L and *p* = 0.0012 for VLP-GM-CSF), and 20:1 (*p* = 0.0044 for VLP-CD40L and *p* = 0.0033 for VLP-GM-CSF). Additionally, the specific cytotoxic activities were even higher than those of the vaccine group with E/T ratios of 100:1 (*p* = 0.0077 for VLP-CD40L and *p* = 0.0018 for VLP-GM-CSF), 50:1 (*p* = 0.0001 for VLP-CD40L and *p* = 0.0019 for VLP-GM-CSF), and 20:1 (*p* = 0.0078 for VLP-CD40L and *p* = 0.0101 for VLP-GM-CSF). The specific cytotoxic activity of HTNV VLPs with added soluble CD40L or GM-CSF was not significantly higher than the vaccine group. In contrast, the non-specific cytotoxicity in the naive group was extremely weak at E/T ratios of 100:1, 50:1, and 20:1 (Figure 10C). The results were similar when purified HTNV GP (Figure 10D) was used for stimulation. Based on these results, CD40L and GM-CSF incorporated into HTNV VLPs induced very specific cytotoxic activity.

Based on these findings, we conclude that CD40L and GM-CSF incorporated into HTNV VLPs elicited enhanced cellular immune responses.

### HTNV VLPs and chimeric HTNV VLPs induced protective immunity against HTNV challenge in C57BL/6 mice

**Figure 11:**
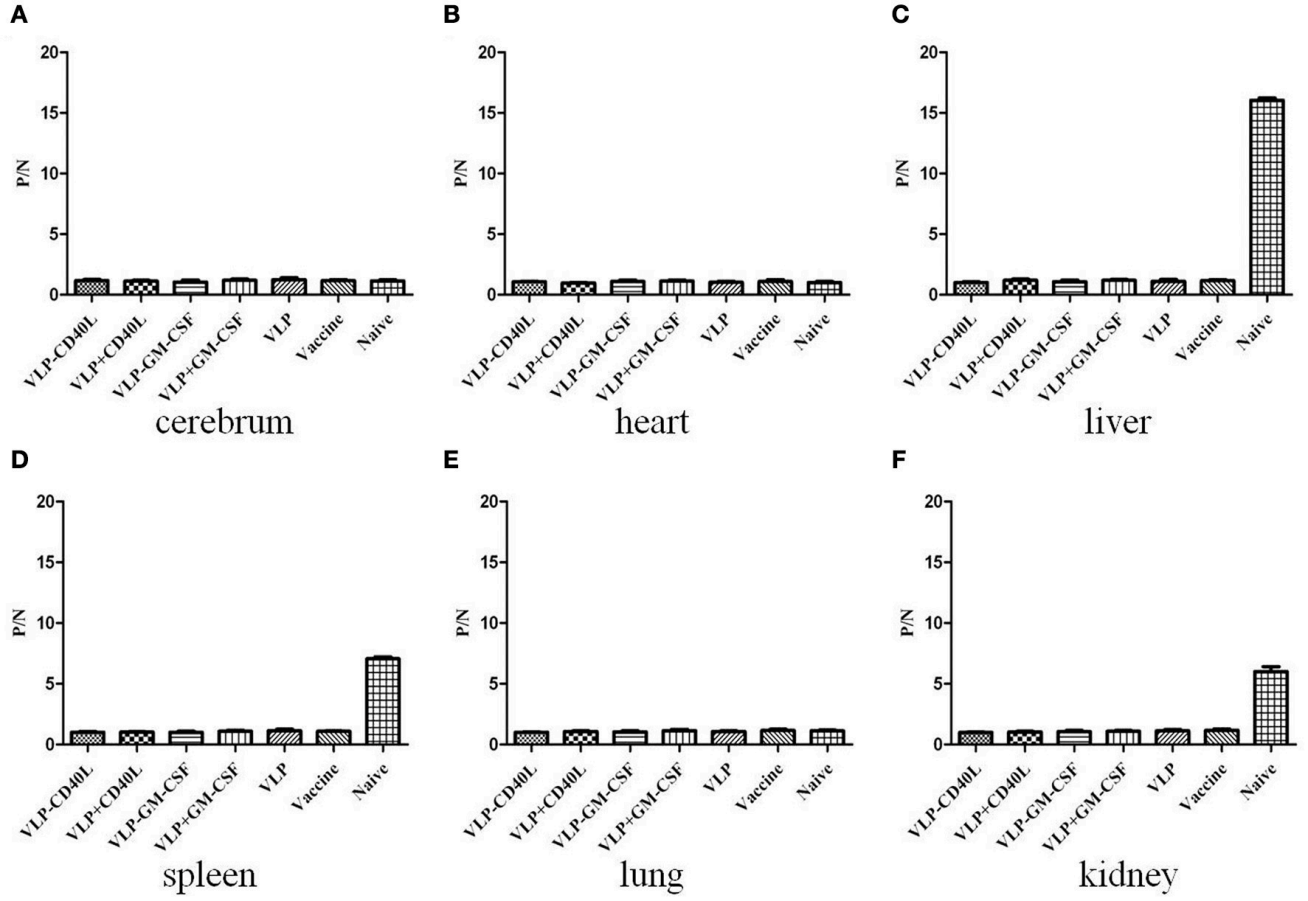
**ELISA detection of HTNV-specific antigens from the major tissues of immunized C57BL/6 mice after being challenged with HTNV**. The mouse tissues were collected as described in the Section Materials and Methods. The results are expressed as the mean value ± SD of four independent experiments. VLP-GM-CSF or VLP-CD40L indicates the HTNV chimeric VLPs, VLP+GM-CSF, or VLP+CD40L indicates the HTNV VLPs with soluble GM-CSF or CD40L. **(A)** Detection of HTNV-specific antigens in the cerebrum. **(B)** Detection of HTNV-specific antigens in the heart. **(C)** Detection of HTNV-specific antigens in the liver. **(D)** Detection of HTNV-specific antigens in the spleen. **(E)** Detection of HTNV-specific antigens in the lungs. **(F)** Detection of HTNV-specific antigens in the kidneys.

The mice were challenged with HTNV 10 d after the final booster immunization to assess protective immunity. HTNV-specific antigens in the tissues of mice were first detected after 3 d using ELISA. We found that the HTNV-specific antigens were detected in the livers, spleens, and kidneys of C57BL/6 mice in the naive group (P/N = 16.05, 7.06, and 6.02, respectively). However, HTNV-specific antigens were not detected in the livers, spleens, and kidneys of C57BL/6 mice in any of the experimental groups or the inactivated Hantavirus vaccine group (Figures 11C,D,F). HTNV-specific antigens were not detected in other tissues of C57BL/6 mice in any of the groups (Figures 11A,B,E). These results indicated that the immunization of C57BL/6 mice with HTNV VLPs conferred protective immunity against HTNV challenge.

**Figure 12:**
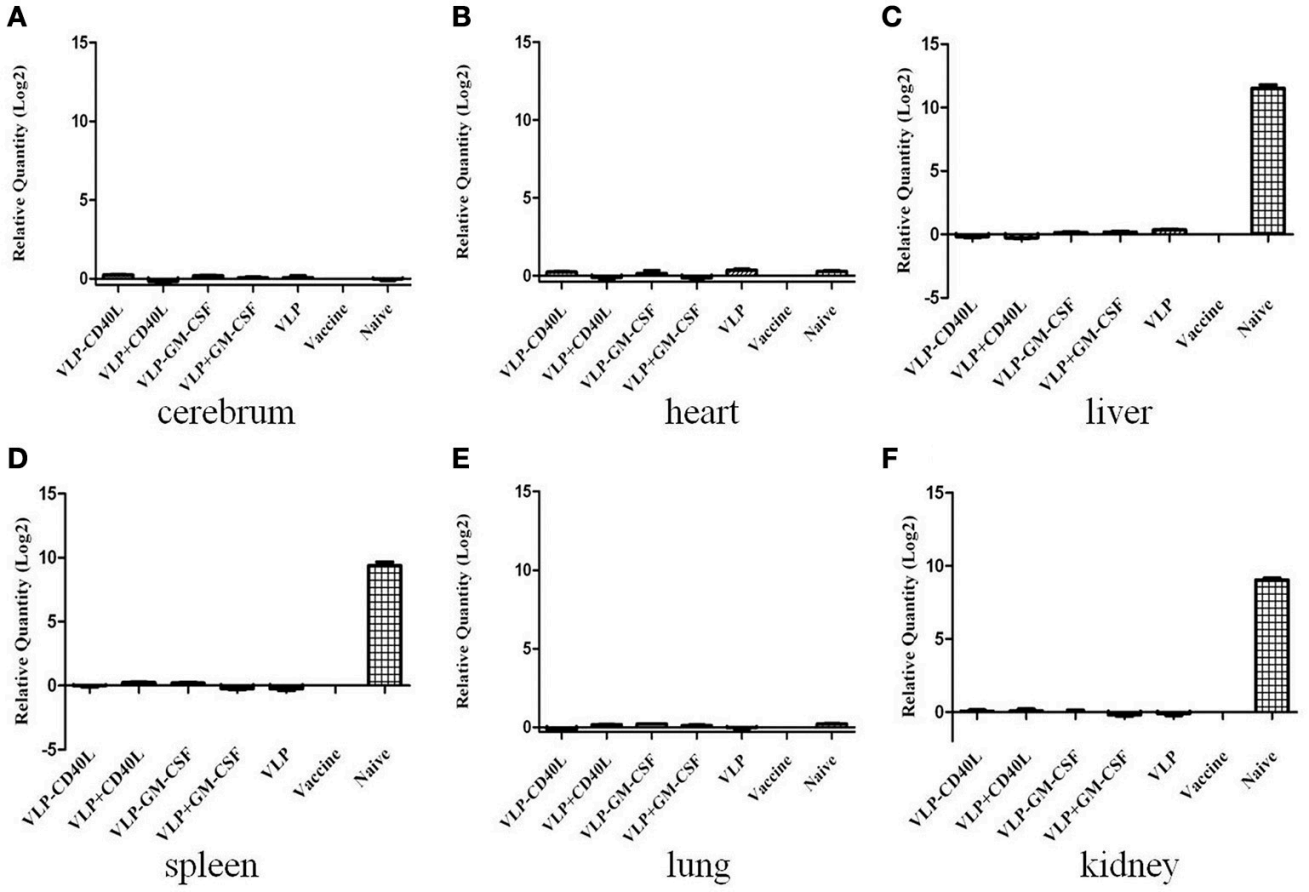
**qRT-PCR detection of HTNV-specific nucleic acids from the major tissues of immunized C57BL/6 mice after being challenged with HTNV**. The mouse tissues were collected as described in the Section Materials and Methods. The results are expressed as the mean value ± SD of four independent experiments. VLP-GM-CSF or VLP-CD40L indicates the HTNV chimeric VLPs, VLP+GM-CSF, or VLP+CD40L indicates the HTNV VLPs with soluble GM-CSF or CD40L. **(A)** Detection of HTNV-specific nucleic acids in the cerebrum. **(B)** Detection of HTNV-specific nucleic acids in the heart. **(C)** Detection of HTNV-specific nucleic acids in the liver. **(D)** Detection of HTNV-specific nucleic acids in the spleen. **(E)** Detection of HTNV-specific nucleic acids in the lungs. **(F)** Detection of HTNV-specific nucleic acids in the kidneys.

Using qRT-PCR, we also detected HTNV-specific nucleic acids in the tissues of immunized C57BL/6 mice after challenge with HTNV. We found that HTNV-specific nucleic acids were detected in the livers, spleens and kidneys of C57BL/6 mice in the naive group, with 2^11.5^-, 2^9.37^- and 2^9.03^-fold amplification, respectively, relative to the inactivated Hantavirus vaccine group. However, HTNV-specific nucleic acids were not detected in the livers, spleens and kidneys of C57BL/6 mice in any of the experimental groups or the inactivated Hantavirus vaccine group (Figures 12C,D,F). Moreover, HTNV-specific nucleic acids were not detected in any other tissues of the C57BL/6 mice in any of the groups (Figures 12A,B,E). These results were consistent with the ELISA results.

Taken together, these results demonstrate that HTNV VLPs and chimeric HTNV VLPs induced protective immunity in C57BL/6 mice against HTNV challenge.

## Discussion

This study is the first to demonstrate that membrane-bound forms of the immunostimulatory molecules GM-CSF and CD40L could be incorporated into HTNV VLPs in a functionally active form to enhance immune responses in C57BL/6 mice when expressed in insect cells coinfected with rBVs expressing HTNV GP, NP, and GPI-anchored GM-CSF or CD40L. Furthermore, we demonstrated that these GM-CSF and CD40L molecules maintained their biological activities when incorporated into VLPs and that immunization with chimeric HTNV VLPs protected C57BL/6 mice against HTNV challenge.

### In the case of the baculovirus expression system (BEVS), the MOI, TOI, and CCI were used to regulate VLP production

In the case of the BEVS, the following three key parameters were used to regulate VLP production: the MOI (Aucoin et al., 2006; Mena et al., 2010), the time of infection (TOI; Palomares et al., 2002), and the cell concentration at the time of infection (CCI; Maranga et al., 2004). In the present study, the HTNV VLPs and chimeric HTNV VLPs were produced using a coinfection strategy. The use of coinfection in this study has allowed combinatorial choices of the MOI ratios, TOI, and CCI. We experimented with many different combinations of MOI ratios, TOI, and CCI (Table 1) to produce the HTNV VLPs and chimeric HTNV VLPs and finally chose to coinfect Sf9 cells with rBV-M and rBV-S at an MOI ratio of 1:1 and rBV-M, rBV-S, and rBV-GM-CSF or rBV-CD40L at a MOI ratio of 1:1:3. The overall MOI was 20 PFU/cell, the TOI was 1 h after the inoculation of Sf9 insect cells (early phase), and the CCI was 1 × 10^7^ cells/ml (high concentration). The incorporation of either GM-CSF or CD40L into HTNV VLPs with a higher MOI of rBV-GM-CSF or rBV-CD40L would be advantageous. The high overall MOI and CCI were used to ensure that the peak cell density was reached when all of the cells were infected and that every cell was coinfected with all of the rBVs (Wong et al., 1996). The early TOI was used to ensure sufficient nutrient levels in the culture medium and determine the cell state that would be advantageous for increasing VLP production. A late TOI could be used unless the culture medium was replaced at the time of infection (Sokolenko et al., 2012). Because the viral infection was similar to a random Poisson process (Kamen et al., 1996; Hu and Bentley, 2001; Palomares et al., 2002), we could not ensure that all of the VLPs produced were correctly assembled using the above strategies. Increasing the ratio of the correctly assembled VLPs and purifying these VLPs using sucrose gradient ultracentrifugation would be beneficial. These results confirmed that HTNV VLPs and chimeric HTNV VLPs were successfully produced.

**Table 1:** **Different combinations of MOI ratios, TOI, and CCI used to produce the HTNV VLPs and chimeric HTNV VLPs**.

**MOI ratios**		**Overall MOI**	**TOI**	**CCI**
rBV-M: rBV-S	1:0.1 1:1 1:10 1:1:0.1	40 PFU/cell 20 PFU/cell 10 PFU/cell	1 h after the inoculation of Sf9 insect cells (early phase); 24 h after the inoculation of Sf9 insect cells (late phase)	1 × 10^7^ cells/ml (high concentration) 1 × 10^6^ cells/ml (medium concentration)
rBV-M: rBV-S:	1:1:1	5 PFU/cell		
rBV-CD40L or	1:1:3	1 PFU/cell		1 × 10^5^ cells/ml (low concentration)
rBV-GM-CSF	1:1:5 1:1:10	0.1 PFU/cell		

*Final choice: MOI ratios: rBV-M, rBV-S: 1:1; rBV-M, rBV-S, rBV-CD40L or rBV-GM-CSF: 1:1:3; Overall MOI: 20 PFU/cell; TOI: 1 h after the inoculation of Sf9 insect cells (early phase); CCI: 1 × 10^7^ cells/ml (high concentration)*.

### Incorporation of CD40L into HTNV VLPs enhanced their immunogenicity

The present study demonstrated that the incorporation of CD40L into HTNV VLPs enhanced their immunogenicity, as evidenced by the induction of both humoral and cellular immune responses against HTNV NP and GP *in vivo*. The augmented immunogenicity may have resulted from the enhanced ability of HTNV VLP-CD40L to induce the phenotypic and functional maturation of DCs. CD40L binding to CD40 on the DCs increases the expression of surface MHC, adhesion and costimulatory molecules, facilitating antigen processing, and presentation by MHC class II molecules to CD4^+^ T cells (Svensson et al., 1997). IL-12 is upregulated in DCs activated by CD40L binding (Yellin et al., 1995; Van Kooten et al., 2000), IL-12 is a cytokine responsible for polarizing CD4^+^ T cells to adopt a Th1 phenotype that primarily secretes IFN-γ and IL-2 and enhancing CD8^+^ T cell proliferation. Polarizing CD4^+^ T cells to adopt a Th1 phenotype generally inhibits the Th2 phenotype that primarily secretes IL-4 and IL-10 (Pulendran et al., 1999). In the present study, we found that HTNV VLP-CD40L increased the numbers of DC1 cells for polarizing CD4^+^ T cells to adopt a Th1 phenotype (Figure 8C), we also found that HTNV VLP-CD40L produced higher levels of IFN-γ and IL-2 compared to HTNV VLPs; however, the IL-4 and IL-10 levels did not change significantly in any of the immunization groups (Figures 10A,B). These results indicate that the incorporation of CD40L into the HTNV-VLPs enhanced the secretion of IFN-γ and IL-2. In addition, CD40L-activated DCs and other APCs have the ability to present exogenous peptides (VLPs) on MHC class I molecules and increase the stimulation of CD8^+^ T cell responses. Our findings also demonstrate that CD40L incorporation into HTNV VLPs produces strong primary CD4^+^ Th1 and CD8+ T cell responses. Other studies that incorporated CD40L into VLPs to enhance immunogenicity reported similar results (Skountzou et al., 2007; Zhang et al., 2010). The incorporation of CD40L into HTNV VLPs also increased the titers of GP- and NP-specific antibodies and the neutralization antibody. Sufficient amounts of antigen must be present in secondary lymphoid organs to stimulate B cells in the marginal zone to induce specific antibody responses. A single dose of HTNV VLPs was not sufficient to induce strong humoral immune responses. In contrast, CD40L may enhance the activation levels of marginal zone APCs by binding to CD40 on APCs and thus may provide sufficient activation signals to B cells with a single dose of HTNV VLPs (Schilizzi et al., 1997). In addition, CD40L-activated Th cells also facilitate the proliferation and differentiation of B cells. Thus, the titers of GP- and NP specific antibodies and neutralization antibody induced by HTNV VLP-CD40L were higher relative to those induced by HTNV VLPs.

### Incorporation of GM-CSF into HTNV VLPs enhanced their immunogenicity

The present study also showed that the incorporation of GM-CSF into HTNV VLPs enhanced their immunogenicity, as evidenced by the induction of both humoral and cellular immune responses against HTNV NP and GP *in vivo*. Similar to CD40L, GM-CSF is an important hematopoietic growth factor and immune modulator that strongly stimulates the proliferation and maturation of DCs, which are crucial in inducing T cell-mediated and B cell-mediated responses (Metcalf, 1986; Gasson, 1991). Moreover, GM-CSF can attract APCs toward the antigens and allows for possible interactions with APCs to enhance uptake (Morrissey et al., 1987). We demonstrated that the incorporation of GM-CSF into HTNV VLPs can induce the proliferation of bone marrow cells and can generate DCs. The DCs produced appear to be of myeloid origin (CD11c^+^, CD11b^+^), which is consistent with previous data for GM-CSF-derived DCs.

### Incorporation of CD40L and GM-CSF into HTNV VLPs significantly enhanced their immunogenicity over the inactivated HTNV vaccine

Although, we should not ignore the potential capacity of inactivated HTNV vaccine to protect people from HTNV infection, our results showed that the incorporation of either CD40L or GM-CSF into HTNV VLPs significantly enhanced their immunogenicity over the inactivated HTNV vaccine, particularly the neutralizing antibody titers and the CD8^+^ T cell-mediated immune responses, which should be the primary aims of anti-HTNV vaccination. The following possibilities may explain the observed differences between the chimeric HTNV VLPs and the inactivated vaccine: (i) the inactivated vaccine used in this study without adjuvant might cause the inactivated vaccine to induce weak specific immune responses; (ii) VLP antigens can be produced to present antigens through the major MHC II exogenous pathway and the MHC I endogenous pathway, inducing both CD4^+^ and CD8^+^ T cell-mediated immune responses, however, the inactivated vaccine mainly presents antigens through the MHC II pathway which induces CD4^+^ T cell-mediated immune responses. Our results also showed that the incorporation of CD40L and GM-CSF into HTNV VLPs significantly enhanced their immunogenicity over HTNV VLPs with added soluble CD40L or GM-CSF. We proposed that chimeric HTNV VLPs may efficiently attract APCs toward the HTNV VLPs and allow for possible interactions with APCs to enhance uptake. However, we could not ignore the capacity of the adjuvant from soluble CD40L or GM-CSF because these molecules facilitated the enhancement of immunogenicity by HTNV VLPs more efficiently than HTNV VLPs alone.

### The development of HFRS animal models has been an intense area of research

In HFRS vaccine research, animal protection experiments represent an important indicator of the effectiveness of a vaccine. The selection of the most appropriate animal model is critical for animal protection experiments. The development of HFRS animal models has been intensely researched. Currently, there are no animal models that reflect the disease manifestations of severe HFRS. Non-human primates often provide good models for studying hantaviruses and therefore have been assessed as potential HFRS models, although with limited success (Yanagihara et al., 1988; Gowen and Holbrook, 2008). Several small laboratory animals have been experimentally infected with HTNV species, resulting in the characterization of traditional infection and/or acute disease models. Perhaps the most successful of these potential models involve the infection of newborn (suckling; McKee et al., 1985; Ebihara et al., 2000) and juvenile mice (Seto et al., 2012) with HTNV usually producing the lethal form of the disease depending on the virus strain and route of infection utilized. However, we are also aware that newborn and juvenile mice are not immunocompetent and are not suitable for animal protection experiments with vaccines. In the present study, we found that HTNV-specific antigens and nucleic acids could only be detected in the livers, spleens and kidneys of C57BL/6 mice in naive groups but not in the other groups 3 days after infection with the HTNV 76–118 strain via intramuscular injection. Further studies are required to better understand why the distributions of HTNV-specific antigens and nucleic acids were primarily localized in the livers, spleens, and kidneys rather than other tissues of the C57BL/6 mice. These observations indicated that C57BL/6 mice immunized with VLPs or vaccine were protected from infection with the HTNV 76–118 strain. According to our observations, VLPs may be a new approach for animal protection experiments for the HTNV vaccine.

In summary, the results presented in the current study demonstrated that the immunostimulatory molecules GM-CSF and CD40L could be incorporated into HTNV VLPs in their functionally active forms, resulting in the enhancement of the immunogenicity of HTNV antigens. We found that GM-CSF and CD40L incorporation into HTNV VLPs induced significantly high levels of humoral and cellular immune responses and protected C57BL/6 mice against HTNV challenge. This study demonstrates that the surfaces of VLPs can be coated with biologically active molecules and that HTNV VLP-GM-CSF and HTNV VLP-CD40L could be utilized as a new type of HTNV vaccine. Further studies are required to better understand the process of incorporation, to increase the levels of incorporation into VLPs and determine how this incorporation affects the immune responses produced.

## Author contributions

LC, FW, LZ, LY, and WY are responsible for performance of experiments. ZL and QY are responsible for collecting data. FZ, ZX, and XW are responsible for experimental design. LC and FW are responsible for writing the manuscript. All authors read and approved the final manuscript.

### Conflict of interest statement

The authors declare that the research was conducted in the absence of any commercial or financial relationships that could be construed as a potential conflict of interest.
